# Relationship of Stress Test Findings to Anatomic or Functional Extent of Coronary Artery Disease Assessed by Coronary Computed Tomography Angiography-Derived Fractional Flow Reserve

**DOI:** 10.1155/2021/6674144

**Published:** 2021-02-24

**Authors:** Demetrios Doukas, Sorcha Allen, Amy Wozniak, Siri Kunchakarra, Rina Verma, Jessica Marot, John J. Lopez, Koen Nieman, Gianluca Pontone, Jonathon Leipsic, Jeroen Bax, Mark G. Rabbat

**Affiliations:** ^1^Division of Cardiology, Loyola University Medical Center, Maywood, IL 60153, USA; ^2^Department of Medicine, Loyola University Medical Center, Maywood, IL 60153, USA; ^3^Division of Cardiology, UCSF Fresno Medical Education Program, Fresno, CA 93701, USA; ^4^Division of Cardiology, Alexian Brothers Medical Center, Elk Grove Village, IL 69997, USA; ^5^Department of Cardiovascular Medicine and Radiology, Stanford University, Stanford, CA, USA; ^6^Department of Cardiovascular Imaging, Cardiologico Monzino, Via Carlo Parea, 4, 20138 Milan, Italy; ^7^Department of Radiology, St. Paul's Hospital and the University of British Columbia, Vancouver, BC, Canada V6T 1Z4; ^8^Department of Cardiology, Leiden University Medical Center, Albinusdreef 2, 2333 ZA Leiden, Netherlands

## Abstract

**Background:**

In the United States, functional stress testing is the primary imaging modality for patients with stable symptoms suspected to represent coronary artery disease (CAD). Coronary computed tomography angiography (CTA) is excellent at identifying anatomic coronary artery disease (CAD). The application of computational fluid dynamics to coronary CTA allows fractional flow reserve (FFR) to be calculated noninvasively (FFR_CT_). The relationship of noninvasive stress testing to coronary CTA and FFR_CT_ in real-world clinical practice has not been studied.

**Methods:**

We evaluated 206 consecutive patients at Loyola University Chicago with suspected CAD who underwent noninvasive stress testing followed by coronary CTA and FFR_CT_ when indicated. Patients were categorized by stress test results (positive, negative, indeterminate, and equivocal). Duke treadmill score (DTS), METS, exercise duration, and chest pain with exercise were analyzed. Lesions ≥ 50%stenosis were considered positive by coronary CTA. FFR_CT_ < 0.80 was considered diagnostic of ischemia.

**Results:**

Two hundred and six patients had paired noninvasive stress test and coronary CTA/FFR_CT_ results. The median time from stress test to coronary CTA was 49 days. Average patient age was 60.3 years, and 42% were male. Of the 206 stress tests, 75% were exercise (70% echocardiographic, 26% nuclear, and 4% EKG). There were no associations of stress test results with CAD > 50% or FFR_CT_ < 0.80 (*p* = 0.927 and *p* = 0.910, respectively). Of those with a positive stress test, only 30% (3/10) had CAD > 50% and only 50% (5/10) had FFR_CT_ < 0.80. Chest pain with exercise did not correlate with CAD > 50% or FFR_CT_ < 0.80 (*p* = 0.66 and *p* = 0.12, respectively). There were no significant correlations between METS, DTS, or exercise duration and FFR_CT_ (*r* = 0.093, *p* = 0.274; *r* = 0.012, *p* = 0.883; and *r* = 0.034, *p* = 0.680; respectively).

**Conclusion:**

Noninvasive stress testing, functional capacity, chest pain with exercise, and DTS are not associated with anatomic or functional CAD using a diagnostic strategy of coronary CTA and FFR_CT_.

## 1. Introduction

In the United States, functional stress testing is the primary imaging modality for patients with stable symptoms suspected to represent coronary artery disease (CAD). Metrics of functional capacity derived from stress tests such as exercise duration, metabolic equivalents (METS), and Duke treadmill score (DTS), an index that provides information calculated using data from exercise treadmill EKG, are commonly reported and incorporated in clinical decision-making to determine the presence of CAD [1]. However, functional stress testing has been shown to have low diagnostic yield at the time of ICA and, consequently, is no longer recommended as the first line diagnostic testing in the National Institute for Health and Care Excellence (NICE) guidelines for the assessment of recent onset chest pain [2]. The necessity of improved methods for the noninvasive evaluation of CAD was highlighted in a retrospective study of the National Cardiovascular Data Registry, which demonstrated that only 37.6% of the 398,978 patients without known CAD who underwent ICA had obstructive CAD, and having a positive noninvasive stress test only increased the rate of obstructive disease from 35% to 41% [3].

Coronary computed tomographic angiography (CTA) has emerged as an excellent noninvasive test for detecting CAD. However, the identification of CAD alone is insufficient as the relationship between coronary stenosis and ischemia is complex and frequently discordant. Over the past few years, there has been strong interest in computing fractional flow reserve (FFR) noninvasively using coronary CTA [4]. The application of computational fluid dynamics (CFD) to resting coronary CTA datasets allows FFR to be calculated noninvasively (FFR_CT_). The emergence of FFR_CT_ provides a noninvasive test that yields both anatomic and functional data and has been validated through a number of accuracy studies [5, 6]. Furthermore, several studies now suggest that FFR_CT_ leads to the reduction of unnecessary ICA in patients with CAD [7–9].

We sought to determine the relationship between noninvasive stress testing, metrics of functional capacity, DTS, and chest pain with exercise and anatomic or functional CAD using a diagnostic strategy of coronary CTA and FFR_CT_.

## 2. Methods

### 2.1. Study Population

We retrospectively evaluated 597 consecutive patients at Loyola University Chicago with suspected CAD who underwent coronary CTA at the treating physician's discretion. Patients with known CAD were excluded from the analysis, and no patients underwent revascularization between stress testing and coronary CTA. Of those patients, 206 had paired noninvasive stress testing and coronary CTA/FFR_CT_ and were included in the analysis. The median time between coronary CTA and stress testing was 49 days.

Due to the retrospective nature of this study, the ordering physicians were not blinded to the results of either the coronary CTA or noninvasive stress test. The coronary CTA studies were read by cardiology attendings with board certification in cardiovascular CT imaging, with support from diagnostic radiology for extracardiac pathology. Exercise treadmill EKGs and stress echocardiograms (exercise and pharmacological) were read by cardiology attendings with board certification in echocardiography. Nuclear stress tests were interpreted by nuclear medicine attendings with board certification in nuclear cardiology.

Coronary artery lesions with ≥50% stenosis were considered positive by coronary CTA whereas FFR_CT_ ≤ 0.80 at the distal vessel tip was considered diagnostic of ischemia. Modalities of noninvasive stress testing included exercise treadmill EKG, stress echocardiogram (exercise and pharmacological), and single-photon emission computed tomography myocardial perfusion imaging (SPECT-MPI [exercise and pharmacological]). Patients were categorized by stress test results (positive, negative, indeterminate, and equivocal). The definition of a positive stress test depended on the stress modality and is described in detail for each below. Patients with discordant stress EKG compared with stress imaging were considered to have equivocal stress tests (i.e., abnormal stress EKG but normal stress echocardiographic images). Indeterminate stress tests were defined as patients who failed to achieve target heart rate or had uninterpretable exercise stress imaging.

### 2.2. Exercise Treadmill EKG

A symptom-limited standard exercise treadmill test (ETT) was conducted, using the Bruce or modified-Bruce protocol. Patients with the following resting EKG changes were excluded: preexcitation (Wolff-Parkinson-White) syndrome, electronically paced ventricular rhythm, greater than 1 mm of resting ST depression, or complete left bundle branch block. The test was preceded by 48-hour discontinuation of *β*-blockers, calcium antagonists, and long-lasting nitrates. The patients were monitored continuously during the test with 12-lead EKG. Exercise duration, METS, chest pain during exercise, arrhythmia, and hypertensive response with stress and ST segment changes were recorded. A positive exercise treadmill EKG was defined as greater than or equal to 1 mm of horizontal or downsloping ST-segment depression or elevation for at least 60 to 80 milliseconds after the end of the QRS complex in 2 or more contiguous leads [10]. Arrhythmia that occurred during exercise included premature ventricular contractions, ventricular tachycardia/fibrillation, or supraventricular tachycardia. A systolic blood pressure > 220 mmHg for men or >210 mmHg for women was considered a hypertensive response. Duke treadmill score was calculated using the following equation: DTS = exercise time − (5 × ST deviation) − (4 × exercise angina), with 0 = none, 1 = non-limiting, and 2 = exercise limiting angina. Patients were further categorized into low risk (score > 5), intermediate risk (score between 4 and -11), and high risk (score < −11) DTS [11].

### 2.3. Stress Echocardiogram

Stress echocardiograms were performed following the guidelines of the American Society of Echocardiography [11]. For stress echocardiography with treadmill testing, the Bruce protocol was utilized and images were obtained at rest, immediately after peak exercise, and at recovery. The patient exercised at 3-minute stages of progressively increasing difficulty until exercise-limiting symptoms, or significant abnormalities in blood pressure, heart rhythm, or ST segments were noted. Postexercise images were obtained as soon as possible and ideally within 1 minute. An ischemic response to exercise was defined by the development of a new wall motion abnormality in a segment with normal function at rest, worsening of function with stress in a segment with a resting wall motion abnormality, increase in the ventricular cavity size with exercise, or a decrease in the ejection fraction compared with rest [11].

### 2.4. Single-Photon Emission Computed Tomography Myocardial Perfusion Imaging

SPECT-MPI was acquired following the guidelines of the American Society of Nuclear Cardiology [12]. Similar to stress echocardiography, patients who underwent exercise SPECT-MPI followed the Bruce protocol and were continuously monitored during the exercise test and for at least 5 minutes into the recovery phase. A 12-lead EKG was obtained at every stage of exercise, at peak exercise, and at the termination or recovery phase. The heart rate and blood pressure were recorded at least every 3 minutes during exercise, at peak exercise, and for at least 5 minutes into the recovery phase. The radiopharmaceutical was injected as close to peak exercise as possible. An abnormal response to stress was a perfusion defect within one or more of the 17-segment heart model territories compared to rest. In addition, an increase in the ventricular cavity with stress was considered an abnormal ischemic response.

### 2.5. Coronary CTA Acquisition and Analysis

Coronary CTA was performed with electrocardiographic gated prospective or retrospective gating on ≥64 detector row scanners (Siemens Sensation Cardiac 64, Siemens Medical Solutions, Malvern, Pennsylvania; Discovery HD 750, GE Healthcare, Milwaukee, USA; Revolution CT 256-row, GE Healthcare, Milwaukee, USA) in accordance with the Society of Cardiovascular Computed Tomography (SCCT) guidelines [13]. Oral, and when needed, intravenous beta-blocker was administered to achieve a target heart rate (HR) of 60 beats per minute (bpm). Sublingual nitroglycerin 0.4-0.8 mg was given approximately 5 minutes prior to contrast administration. CTA datasets were interpreted using a commercially available dedicated workstation (Aquarius 3D Workstation, TeraRecon, San Mateo, CA, USA). A coronary lesion with ≥50% diameter of stenosis by the interpreting physician was considered obstructive on coronary CTA [14–16]. Coronary vessel branches for the left anterior descending, left circumflex, and right coronary arteries were categorized according to the SCCT guidelines.

### 2.6. Computation of FFR_CT_

FFR_CT_ analysis was performed by HeartFlow Inc. (Redwood City, California) as previously described [17]. After semiautomated segmentation of the epicardial coronary arteries and determination of left ventricular mass, calculations of FFR_CT_ were performed by CFD modeling. Three-dimensional (3D) blood flow modeling of the coronary arteries was performed, with blood modeled as a Newtonian fluid using incompressible Navier–Stokes equations and solved subject to appropriate initial and boundary conditions using a finite element method on a parallel supercomputer. Coronary blood flow was simulated under conditions modeling intravenous adenosine-mediated coronary hyperemia. A positive FFR_CT_ was defined as the distal tip value < 0.80 in a vessel of diameter > 1.8 mm.

### 2.7. Statistical Analysis

Baseline characteristics of the selected subjects were calculated and presented as frequencies and percentages for categorical variables and mean ± SD for continuous variable. General descriptive statistics (means, standard deviations, and frequencies) were used to summarize patient characteristics and stress-test results for the entire cohort and separately for each group. Student's *t*-test were used to compare associations of continuous variables, and chi-sq test or Fisher's exact test was used to compare associations of categorical variables. Pearson's correlation coefficients estimated correlation between continuous predictors and continuous FFR-CT. All analyses were performed using SAS Proprietary software (version 9.2, SAS Institute, Cary, North Carolina).

## 3. Results

206 patients had a noninvasive stress test and coronary CTA/FFR_CT_ result. Using the Diamond–Forrester score, 86.1% of patients were at an intermediate clinic risk. Associations between clinical characteristics, functional capacity, stress test findings, and FFR_CT_ results with CAD > 50% are outlined in Table 1. The average patient age was 60.3 years, and 42% of the cohort were male. The average patient BMI was 29.5 kg/m^2^. Older age, hypertension, hyperlipidemia, and FFR_CT_ < 0.80 were all significantly associated with CAD > 50%. Arrhythmia and hypertensive response with stress, DTS, METS, and exercise duration were not associated with CAD > 50% (*p* = 0.66, *p* = 0.70, *p* = 0.59, *p* = 0.07, and *p* = 0.25, respectively). Furthermore, the development of chest pain during exercise did not correlate with CAD > 50% (*p* = 0.66).

Table 2 outlines clinical characteristics, functional capacity, stress test findings, and the association with FFR_CT_. Hyperlipidemia was associated with positive FFR_CT_ (*p* = 0.007, Table 2). Arrhythmia and hypertensive response with stress, DTS, METS, and exercise duration were not associated with positive FFR_CT_ (*p* = 0.56, *p* = 0.53, *p* = 0.30, *p* = 0.90, and *p* = 0.54, respectively). Development of chest pain during the stress test was not associated with positive FFR_CT_ (*p* = 0.121, Table 2).

**Figure 1:**
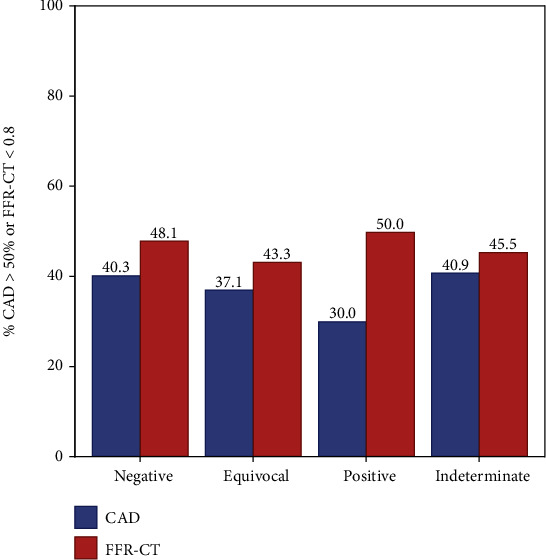
Percentage of CAD > 50% or FFR-CT < 0.80 by stress test result.

**Figure 2:**
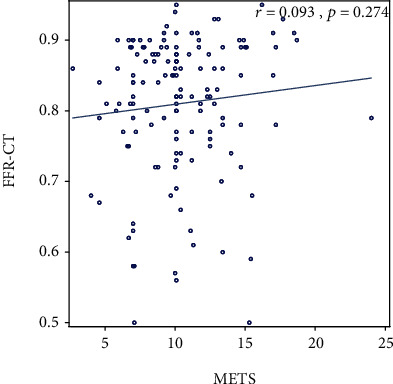
Scatter plot showing correlation between metabolic equivalents achieved and FFR_CT_.

**Figure 3:**
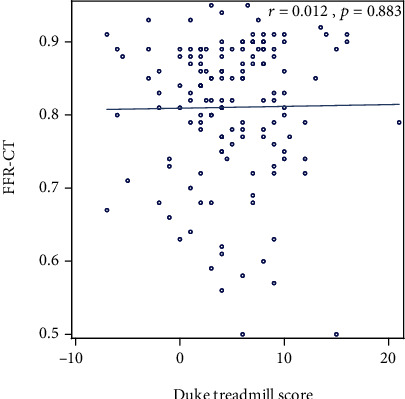
Scatter plot showing correlation between Duke treadmill score and FFR_CT_.

**Figure 4:**
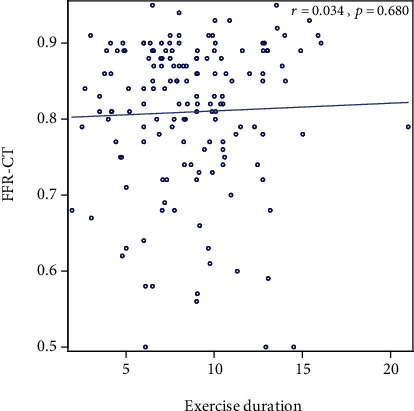
Scatter plot showing correlation between exercise duration and FFR_CT_.

Of the 206 stress tests performed, 75% were exercise (70% echocardiographic, 26% nuclear, and 4% EKG alone). Thirty-four percent of patients had an abnormal ETT with ≥1 mm ST depression, but this was not associated with anatomic or functional CADon CTA and FFR_CT_ (*p* = 0.12 and *p* = 0.20, respectively). There was no association between stress test results (positive, negative, equivocal, or indeterminate) and positive CAD > 50% (*p* = 0.91) or FFR_CT_ < 0.80 (*p* = 0.927) (Table 3, Figure 1). Of those with a positive stress test, only 30% (3/10) had CAD > 50% and only 50% (5/10) had FFR_CT_ < 0.80 (*p* = 0.910 and *p* = 0.927, respectively). Of those with a negative stress test, 40% (31/77) had CAD > 50% and 48% (37/77) had FFR_CT_ < 0.80 (*p* = 0.910 and *p* = 0.927, respectively). There was no significant correlation between METS, DTS, or exercise duration and FFR_CT_ (*r* = 0.093, *p* = 0.274; *r* = 0.012, *p* = 0.883; *r* = 0.034, *p* = 0.680, respectively) (Figures 2–4). Tables 1 and 2 in the supplementary section outline patient characteristics and stress test findings stratified by CAD severity ranges.

## 4. Discussion

We identified a number of important findings:

1. In this real-world clinical cohort, positive stress testing in patients without known CAD was not associated with anatomic or functional CAD using a diagnostic strategy of coronary CTA and FFR_CT_
2. Exercise duration, exercise capacity/achieved workload, and DTS were not correlated with anatomic or functional CAD
3. There was no association between chest pain with exercise and anatomic and functional CAD
4. Coronary CTA and FFR_CT_ identified CAD in at-risk patients with equivocal stress tests

For over four decades, functional stress testing has served as the standard cardiovascular diagnostic pathway for those with stable symptoms suggestive of CAD, although it has been reported to have low diagnostic yield at the time of ICA with approximately two-thirds of patients with a positive stress test having no obstructive CAD and 28% of patients with a negative stress test having CAD [3]. An analysis from more than 385,000 patients from >1100 United States hospitals noted that less than half of patients undergoing exercise-treadmill testing, stress echocardiography, and SPECT imaging, prior to their ICA, were found to have obstructive CAD [18]. Noninvasive testing made a similar prediction of obstructive CAD compared to clinical factors. In addition, a Duke University study of over 15,000 patients found that among patients referred for ICA, those with a positive stress test were less likely to have obstructive CAD compared to those with either a negative stress test or no testing at all [19]. Recently, the NIH-funded international ISCHEMIA trial demonstrated that in patients with moderate-severe ischemia on functional stress testing, over 14% demonstrated no obstructive CAD on coronary CTA [20]. Coronary CTA has become an established diagnostic modality for the assessment of CAD [14–16, 21]. It is a sensitive study, reliably confirms the absence of CAD, and aids in the identification of nonobstructive CAD for which providers can institute optimal medical therapy to reduce cardiac events [22]. In the multicenter randomized controlled trial SCOT-HEART, the use of coronary CTA in addition to standard care in patients with stable chest pain resulted in a significantly lower rate of death from heart disease or nonfatal myocardial infarction (MI) than standard care alone [23]. Similar to prior studies, in our analysis, stress testing positivity did not accurately identify obstructive CAD. Only 30% of patients with a positive stress test had obstructive CAD. In addition, of those with a negative stress test, 40% had obstructive CAD.

Similar to ICA, coronary CTA alone does not allow for the interpretation of functional importance of intermediate stenoses. It is well known that there is poor correlation between the angiographic severity of a coronary stenosis and its functional significance and numerous studies have shown that FFR is better at identifying lesions responsible for ischemia and improves outcomes when guiding revascularization [24]. The addition of FFR_CT_ has improved the performance of coronary CTA for the diagnosis of clinically important CAD [5, 25]and decreases the need for ICA [26, 27]. In our analysis, approximately 50% of patients with a negative stress test had a positive FFR_CT_ in at least one epicardial coronary artery. Importantly, only 50% of patients with a positive stress test had a positive FFR_CT_.

Despite its rather low sensitivity for the predication of obstructive CAD, functional capacity, as assessed by ETT, is often regarded as one of the most important prognostic variables [28, 29]. In a seminal work by McNeer et al., patients with poor functional capacity were more likely to have anatomic CAD and worse survival [30]. Whether patients with a high exercise capacity are at a low risk for functional CAD as assessed by FFR_CT_ is unknown. In this analysis, patients had excellent functional capacity, achieving on average 10 METS with a mean exercise duration > 8 minutes. Patients with CAD > 50% had similar functional capacity to those with CAD < 50% disease. Likewise, patients with positive FFR_CT_ had similar achieved workload and exercise duration to those with negative FFR_CT_. In addition, there were no significant correlations between METS, or exercise duration and FFR_CT_.

Although the DTS has been shown to predict adverse outcome and mortality, this analysis did not find an association of DTS with anatomic or functional CAD as assessed by coronary CTA and FFR_CT_. On average, study patients had a low risk DTS. The mean DTS for our cohort was 4.8, with 55% of patients being low annual risk and 45% intermediate risk of death [31]. Although we did not assess mortality, patients with a low DTS may be mistakenly inferred to have nonsignificant CAD translating to a missed opportunity for medical optimization and improved outcomes. Both low and intermediate DTS patients had similar rates of CAD > 50% and/or FFR_CT_ < 0.80, highlighting that the low and intermediate DTS may not be associated with anatomic and functional extent of CAD. Consistent with a prior study using invasive FFR, in our analysis, there was no significant correlation between numerical DTS and FFR_CT_ [32].

Many patients experience MI without any prior symptoms. In a study of over 9000 patients who were free of cardiovascular disease at baseline from the Atherosclerosis Risk in Communities study, >45% of incident MI were asymptomatic in nature [33]. These individuals often lack medical treatments that may prevent subsequent adverse outcomes, including a second MI or even death [34]. In addition, the prognosis of patients with asymptomatic MI is similar, if not worse, than those with clinically evident MI [35]. Various coronary CTA studies in asymptomatic individuals have identified a significant number of patients with prognostically important CAD [36, 37]. Interestingly, in our study, there was no correlation between chest pain during the stress test and anatomic or functional CAD. A coronary CTA and FFR_CT_ diagnostic strategy may play a critical role in identifying and treating these at-risk patients.

Patients with equivocal or discordant stress test findings represent a unique patient population and often present a challenge for the treating physician. In fact, this group represents the largest portion of our stress patients with 47% of patients having an equivocal stress test. Of all the equivocal stress tests, 37% had CAD > 50% and 43% had FFRCT < 0.80. Patients with discordant or equivocal stress results have an excess risk for adverse cardiac events. In a recent large single-center study, researchers analyzed >15,000 patients undergoing stress testing and found that patients with equivocal stress tests had higher rates of major adverse cardiac events compared to patients with negative stress findings [19]. Coronary CTA and FFR_CT_ may play an important role in the diagnosis and management of patients with equivocal stress tests.

## 5. Limitations

Coronary flow reserve has been associated with exercise capacity and was not assessed in this study. Coronary microvascular dysfunction may have been a reason for reduced exercise capacity in patients who have no apparent anatomic or functional epicardial CAD. Stress testing and coronary CTA did not occur on the same day, and it remains possible that CAD could have progressed between study dates. This remains unlikely since there was only a median 49-day difference between study modalities, and no patients underwent revascularization between tests. This is a single-center retrospective study with a limited sample size. Females represented 58% of the study population, which is higher compared to many CAD clinical trials. Recently, FFR_CT_ was noted to differ between sexes as women have a higher FFR_CT_ for the same degree of stenosis [38]. In FFR_CT_-positive CAD, women had less obstructive CAD. Further study is needed comparing gender specific differences of stress test findings to anatomic or functional extent of CAD. In addition, the average BMI of our population was 30 kg/m^2^, which is more typical of the United States population compared to individuals in other geographic areas of the world, and may have impacted our findings. Finally, given the retrospective nature of this study, the choice of stress modality and subsequent referral to CTA is complex for which not all confounding variables can be accounted for and could have led to the potential of inclusion bias. Therefore, the results of this analysis are hypothesis generating and larger analyses are needed to definitively address the association of stress parameters with anatomic and functional epicardial CAD.

## 6. Conclusion

Stress testing results, metrics of functional capacity, chest pain with exercise, and low-intermediate DTS are not associated with anatomic or functional CAD by coronary CTA and FFR_CT_.

## Figures and Tables

**Table 1:** Patient characteristics and associations with CAD > 50%.

Patient characteristics	Total, *N* = 206, *n* (%)	CAD > 50%, *N* = 79, *n* (%)	CAD < 50%, *N* = 127, *n* (%)	*p* value^∗^
Age, mean (SD)	60.3 (11.5)	62.9 (11.5)	58.7 (11.2)	0.011
BMI, mean (SD)	29.5 (5.6)	30 (5.4)	29.2 (5.7)	0.316
Male	87 (42)	36 (46)	51 (40)	0.444
Diabetes	38 (18)	19 (24)	19 (15)	0.102
HPL	145 (70)	63 (80)	82 (65)	0.020
HTN	135 (66)	63 (80)	72 (57)	0.001
Chest pain during study	14 (7)	6 (8)	8 (6)	0.660
Arrhythmia^∗∗^	60 (30)	24 (32)	36 (29)	0.658
Hypertensive response	15 (8)	5 (7)	10 (8)	0.699
ST depression ≥ 1 mm	71 (34)	22 (28)	49 (39)	0.115
DTS: intermediate risk	74 (50)	25 (51)	49 (50)	0.907
DTS: low risk	73 (50)	24 (49)	49 (50)	
Duke treadmill score, mean (SD)	4.8 (4.8)	4.5 (4.7)	5 (4.9)	0.590
METS score, mean (SD)	10.3 (3.4)	9.6 (3.4)	10.7 (3.4)	0.065
Exercise duration	8.6 (3.3)	8.2 (3.3)	8.9 (3.3)	0.249
*FFR-CT:*				
FFR-CT < 0.80	94 (46)	54 (68)	40 (31)	<0.001

^∗^ *p* value calculated with *t*-test, chi-sq test, or Fisher's exact test, where appropriate. ^∗∗^58 PVCs and 2 NSVT/VT.

**Table 2:** Patient characteristics and associations with FFR-CT < 0.80.

Patient characteristics	Total, *N* = 206, *n* (%)	FFR-CT < 0.80, *N* = 94, *n* (%)	FFR-CT > 0.80, *N* = 112, *n* (%)	*p* value^∗^
Age, mean (SD)	60.3 (11.5)	61 (12.3)	59.7 (10.7)	0.421
BMI, mean (SD)	29.5 (5.6)	29.4 (4.9)	29.6 (6.1)	0.782
Male	87 (42)	43 (46)	44 (39)	0.350
Diabetes	38 (18)	20 (21)	18 (16)	0.337
Hyperlipidemia	145 (70)	75 (80)	70 (63)	0.007
HTN	135 (66)	65 (69)	70 (63)	0.317
Chest pain during study	14 (7)	9 (10)	5 (5)	0.121
Arrhythmia^∗∗^	60 (30)	29 (32)	31 (28)	0.563
Hypertensive response	15 (8)	8 (9)	7 (6)	0.526
ST depression ≥ 1 mm	71 (34)	28 (30)	43 (38)	0.196
DTS: intermediate risk	74 (50)	29 (45)	45 (54)	0.284
DTS: low risk	73 (50)	35 (55)	38 (46)	
Duke treadmill score, mean (SD)	4.8 (4.8)	5.3 (5)	4.5 (4.7)	0.297
METS score, mean (SD)	10.3 (3.4)	10.4 (3.6)	10.3 (3.3)	0.902
Exercise duration	8.6 (3.3)	8.8 (3.3)	8.5 (3.2)	0.536

^∗^ *p* value calculated with *t*-test, chi-sq test, or Fisher's exact test, where appropriate. ^∗∗^58 PVCs and 2 NSVT/VT.

**Table 3:** Percentage of CAD > 50% and FFR_CT_ < 0.80 by stress test result.

CAD	Negative *N* = 77	Equivocal *N* = 97	Positive *N* = 10	Indeterminate *N* = 22	*p* value
<50%	46 (59.7%)	61 (62.9%)	7 (70%)	13 (59.1%)	0.910
>50%	31 (40.3%)	36 (37.1%)	3 (30%)	9 (40.9%)	
*FFR_CT_*					
>0.80	40 (51.9%)	55 (56.7%)	5 (50%)	12 (54.5%)	0.927
<0.80	37 (48.1%)	42 (43.3%)	5 (50%)	10 (45.5%)	

## Data Availability

Access to data is available upon request.

## Supplementary Materials

Supplementary Materials Tables 1 and 2 outline patient characteristics and stress test findings stratified by CAD severity ranges.

## References

[1] Mark D. B., Hlatky M. A., Harrell FE Jr, Lee K. L., Califf R. M., Pryor D. B. (1987). Exercise treadmill score for predicting prognosis in coronary artery disease.. *Annals of internal medicine*.

[2] Kelion A. D., Nicol E. D. (2018). The rationale for the primacy of coronary CT angiography in the National Institute for Health and Care Excellence (NICE) guideline (CG95) for the investigation of chest pain of recent onset. *Journal of Cardiovascular Computed Tomography*.

[3] Patel M. R., Peterson E. D., Dai D. (2010). Low diagnostic yield of elective coronary angiography. *New England Journal of Medicine*.

[4] Pontone G., Rabbat M. G. (2017). The New Era of Computational Fluid Dynamics in CT Angiography:. *JACC: Cardiovascular Imaging*.

[5] Koo B. K., Erglis A., Doh J. H. (2011). Diagnosis of ischemia-causing coronary stenoses by noninvasive fractional flow reserve computed from coronary computed tomographic angiograms: results from the prospective multicenter DISCOVER-FLOW (Diagnosis of Ischemia-Causing Stenoses Obtained Via Noninvasive Fractional Flow Reserve) Study. *Journal of the American College of Cardiology*.

[6] Nakazato R., Park H. B., Berman D. S. (2013). Noninvasive fractional flow reserve derived from computed tomography angiography for coronary lesions of intermediate stenosis severity results from the DeFACTO study. *Circulation: Cardiovascular Imaging*.

[7] Douglas P. S., Pontone G., Hlatky M. A. (2015). Clinical outcomes of fractional flow reserve by computed tomographic angiography-guided diagnostic strategies vs. usual care in patients with suspected coronary artery disease: the prospective longitudinal trial of FFRCT: outcome and resource impacts study. *European heart journal*.

[8] Hlatky M. A., de Bruyne B., Pontone G. (2015). Quality-of-life and economic outcomes of assessing fractional flow reserve with computed tomography angiography: PLATFORM. *Journal of the American College of Cardiology*.

[9] Rabbat M., Leipsic J., Bax J. (2020). Fractional flow reserve derived from coronary computed tomography angiography safely defers invasive coronary angiography in patients with stable coronary artery disease. *Journal of Clinical Medicine*.

[10] Fletcher G. F., Ades P. A., Kligfield P. (2013). American Heart Association Exercise, Cardiac Rehabilitation, and Prevention Committee of the Council on Clinical Cardiology, Council on Nutrition, Physical Activity and Metabolism, Council on Cardiovascular and Stroke Nursing, and Council on Epidemiology and Prevention Exercise standards for testing and training. *Circulation*.

[11] Pellikka P. A., Arruda-Olson A., Chaudhry F. A. (2020). Guidelines for performance, interpretation, and application of stress echocardiography in ischemic heart disease: from the American Society of Echocardiography. *Journal of the American Society of Echocardiography*.

[12] Dorbala S., Ananthasubramaniam K., Armstrong I. S. (2018). Single photon emission computed tomography (SPECT) myocardial perfusion imaging guidelines: instrumentation, acquisition, processing, and interpretation. *Journal of Nuclear Cardiology*.

[13] Leipsic J., Abbara S., Achenbach S. (2014). SCCT guidelines for the interpretation and reporting of coronary CT angiography: a report of the Society of Cardiovascular Computed Tomography Guidelines Committee. *Journal of Cardiovascular Computed Tomography*.

[14] Budoff M. J., Dowe D., Jollis J. G. (2008). Diagnostic Performance of 64-Multidetector Row Coronary Computed Tomographic Angiography for Evaluation of Coronary Artery Stenosis in Individuals Without Known Coronary Artery Disease: Results From the Prospective Multicenter ACCURACY (Assessment by Coronary Computed Tomographic Angiography of Individuals Undergoing Invasive Coronary Angiography) Trial. *Journal of the American College of Cardiology*.

[15] Meijboom W. B., Meijs M. F. L., Schuijf J. D. (2008). Diagnostic Accuracy of 64-Slice Computed Tomography Coronary Angiography: A Prospective, Multicenter, Multivendor Study. *Journal of the American College of Cardiology*.

[16] Miller J. M., Rochitte C. E., Dewey M. (2008). Diagnostic performance of coronary angiography by 64-row CT. *The New England Journal of Medicine*.

[17] Taylor C. A., Fonte T. A., Min J. K. (2013). Computational fluid dynamics applied to cardiac computed tomography for noninvasive quantification of fractional flow reserve: scientific basis. *Journal of the American College of Cardiology*.

[18] Patel M. R., Dai D., Hernandez A. F. (2014). Prevalence and predictors of nonobstructive coronary artery disease identified with coronary angiography in contemporary clinical practice. *American heart journal*.

[19] Daubert M. A., Sivak J., Dunning A. (2020). Implications of abnormal exercise electrocardiography with normal stress echocardiography. *JAMA internal medicine*.

[20] Maron D. J., Hochman J. S., Reynolds H. R. (2020). Initial invasive or conservative strategy for stable coronary disease. *New England Journal of Medicine*.

[21] Neglia D., Rovai D., Caselli C. (2015). Detection of significant coronary artery disease by noninvasive anatomical and functional imaging. *Circulation: Cardiovascular Imaging*.

[22] Hwang I. C., Jeon J. Y., Kim Y. (2015). Statin therapy is associated with lower all-cause mortality in patients with non-obstructive coronary artery disease. *Atherosclerosis*.

[23] The SCOT-HEART Investigators, Newby D. E., Adamson P. D. (2018). Coronary CT angiography and 5-year risk of myocardial infarction. *The New England Journal of Medicine*.

[24] de Bruyne B., Fearon W. F., Pijls N. H. J. (2014). Fractional flow reserve-guided PCI for stable coronary Artery disease. *New England Journal of Medicine*.

[25] Min J. K., Leipsic J., Pencina M. J. (2012). Diagnostic accuracy of fractional flow reserve from anatomic CT angiography. *Jama*.

[26] Rabbat M. G., Berman D. S., Kern M. (2017). Interpreting results of coronary computed tomography angiography-derived fractional flow reserve in clinical practice. *Journal of Cardiovascular Computed Tomography*.

[27] Ball C., Pontone G., Rabbat M. (2018). Fractional flow reserve derived from coronary computed tomography angiography datasets: the next frontier in noninvasive assessment of coronary artery disease. *BioMed Research International*.

[28] Myers J., Prakash M., Froelicher V., Do D., Partington S., Atwood J. E. (2002). Exercise capacity and mortality among men referred for exercise testing. *The New England Journal of Medicine*.

[29] Hung R. K., al-Mallah M. H., McEvoy J. W. (2014). Prognostic value of exercise capacity in patients with coronary artery disease: the FIT (Henry Ford Exercise Testing) project. *Mayo Clinic Proceedings*.

[30] McNeer J. F., Margolis J. R., Lee K. L. (1978). The role of the exercise test in the evaluation of patients for ischemic heart disease. *Circulation*.

[31] Mark D. B., Shaw L., Harrell F. E. (1991). Prognostic value of a treadmill exercise score in outpatients with suspected coronary artery disease. *New England Journal of Medicine*.

[32] Kalaycı S., Kalaycı B., Şahan E., Ayyılmaz A. A. (2017). Association between fractional flow reserve and Duke treadmill score in patients with single-vessel disease. *Kardiologia Polska (Polish Heart Journal)*.

[33] Zhang Z. M., Rautaharju P. M., Prineas R. J. (2016). Race and sex differences in the incidence and prognostic significance of silent myocardial infarction in the Atherosclerosis Risk in Communities (ARIC) study. *Circulation*.

[34] Pride Y. B., Piccirillo B. J., Gibson C. M. (2013). Prevalence, consequences, and implications for clinical trials of unrecognized myocardial infarction. *American Journal of Cardiology*.

[35] Kannel W. B., Abbott R. D. (1984). Incidence and prognosis of unrecognized myocardial Infarction. *New England Journal of Medicine*.

[36] Guaricci A. I., Lorenzoni V., Guglielmo M. (2018). Prognostic relevance of subclinical coronary and carotid atherosclerosis in a diabetic and nondiabetic asymptomatic population. *Clinical cardiology*.

[37] di Cesare E., Patriarca L., Panebianco L. (2018). Coronary computed tomography angiography in the evaluation of intermediate risk asymptomatic individuals. *La radiologia medica*.

[38] Fairbairn T. A., Dobson R., Hurwitz-Koweek L. (2020). Sex Differences in Coronary Computed Tomography Angiography-Derived Fractional Flow Reserve: Lessons From ADVANCE. *JACC: Cardiovascular Imaging*.

