# Supplementary material for: Relationship of Stress Test Findings to Anatomic or Functional Extent of Coronary Artery Disease Assessed by Coronary Computed Tomography Angiography-Derived Fractional Flow Reserve
Source: Biomed Res Int. 2021 Feb 24;2021:6674144. doi: 10.1155/2021/6674144 (PMC7929671; doi:10.1155/2021/6674144)
Supplement: Supplementary Materials — Tables 1 and 2 outline patient characteristics and stress test findings stratified by CAD severity ranges. [file 6674144.f1.docx]

Supplementary Table 1. Patient Characteristics and associations with CAD<50%, 50-69%, 70-89%, and >=90%

| Risk Factor | N(%), All | n (%), CAD <50%, N=127 | n (%), CAD 50%-69%, N=51 | n (%), CAD 70%-89%, N=27 | n (%), CAD >=90%, N=3 | P-Value | |
| --- | --- | --- | --- | --- | --- | --- | --- |
| Age, Median (Q1, Q3) | 62 (52, 69) | 59 (51, 66) | 65 (51, 69) | 69 (57, 70) | 63 (62, 75) | 0.021 | |
| BMI, Median (Q1, Q3) | 29 (25.8, 32.3) | 28.9 (25.2, 31.7) | 29 (26.1, 34.9) | 29.5 (26.5, 33.1) | 24.5 (24.4, 29.3) | 0.388 | |
| Male | 87 (42) | 50 (40) | 24 (47) | 11 (41) | 2 (67) | 0.678 | |
| Diabetes | 38 (18) | 19 (15) | 11 (22) | 7 (26) | 1 (33) | 0.304 | |
| HPL | 145 (70) | 81 (65) | 38 (75) | 23 (85) | 3 (100) | 0.108 | |
| HTN | 135 (66) | 71 (57) | 38 (75) | 23 (85) | 3 (100) | 0.005 | |
| Chest Pain During Study | 14 (7) | 8 (7) | 2 (4) | 4 (15) | 0 (0) | 0.342 | |
| Arrythmia** | 60 (30) | 34 (28) | 16 (33) | 10 (38) | 0 (0) | 0.513 | |
| Hypertensive Response | 15 (8) | 10 (8) | 1 (2) | 3 (12) | 1 (33) | 0.093 | |
| ST Depression >=1mm | 71 (34) | 49 (39) | 11 (22) | 11 (41) | 0 (0) | 0.069 | |
| Duke Treadmill Score, Median (Q1, Q3) | 4 (2, 8) | 4 (2, 8) | 6 (2, 9) | 3 (-2, 8) | 6 (4, 6) | 0.386 | |
| METS Score, Median (Q1, Q3) | 10.1 (7.7, 12.5) | 10.1 (8.3, 12.5) | 10.1 (8, 11.2) | 10 (6.7, 11.9) | 7.1 (6.7, 7.1) | 0.123 | |
| Exercise Duration, Median (Q1, Q3) | | 8.4 (6.5, 10.5) | 8.4 (6.5, 10.5) | 8.6 (7.2, 10.5) | 8 (4.7, 9.2) | 6.1 (4.8, 6.1) | 0.169 |
| **FFR-CT** | |  |  |  |  |  |  |
| Positive FFR-CT | | 94 (46) | 39 (31) | 26 (51) | 26 (96) | 3 (100) | <0.001 |

*P-value calculated with Kruskal-Wallis test or Fisher’s exact test, where appropriate

**58 PVCs, 2 NSVT/VT

Supplementary Table 2. Sensitivity Analysis: Percentage of CAD by Stress Test Result

| **CAD** | **Negative**  **N=77** | **Equivocal**  **N=97** | **Positive**  **N=10** | **Indeterminate**  **N=22** | **P-Value** |
| --- | --- | --- | --- | --- | --- |
| <50% | 45 (58.4%) | 61 (62.9%) | 7 (70.0%) | 12 (54.6%) | 0.1943 |
| 50-69% | 22 (28.6%) | 21 (21.6%) | 1 (10.0%) | 7 (31.8%) |  |
| 70-89% | 9 (11.7%) | 15 (15.5%) | 2 (20.0%) | 1 (4.6%) |  |
| >=90% | 1 (1.3%) | 0 (0.0%) | 0 (0.0%) | 2 (9.1%) |  |
